# Neutrophil-to-lymphocyte ratio (NLR) and platelet-to-lymphocyte ratio (PLR) as independent predictors of outcome in infective endocarditis (IE)

**DOI:** 10.1186/s43044-019-0014-2

**Published:** 2019-09-18

**Authors:** Marwa Sayed Meshaal, Abdo Nagi, Ahmed Eldamaty, Wae’el Elnaggar, Mervat Gaber, Hussien Rizk

**Affiliations:** 1 Cardiovascular Medicine Department, Kasr Al Ainy School of Medicine, Cairo University, Cairo, Egypt; 2 Clinical Pathology Department, Kasr Al Ainy School of Medicine, Cairo University, Cairo, Egypt

**Keywords:** Infective endocarditis (IE), Neutrophil-to-lymphocyte ratio (NLR), Platelet-to-lymphocyte ratio (PLR)

## Abstract

**Background:**

Early and accurate risk assessment is an important clinical demand in patients with infective endocarditis (IE). The neutrophil-to-lymphocyte ratio (NLR) and platelet-to-lymphocyte ratio (PLR) are independent predictors of prognosis in many infectious and cardiovascular diseases. Very limited studies have been conducted to evaluate the prognostic role of these markers in IE.

**Results:**

We analyzed clinical, laboratory, and echocardiographic data and outcomes throughout the whole period of hospitalization for a total of 142 consecutive patients with definitive IE.

The overall in-hospital mortality was 21%. Major complications defined as central nervous system embolization, fulminant sepsis, acute heart failure, acute renal failure, and major artery embolization occurred in 38 (27%), 34 (24%), 32 (22.5%), 40 (28%), and 90 (63.4%) patients, respectively.

The NLR, total leucocyte count (TLC), neutrophil percentage, creatinine, and C-reactive protein (CRP) level obtained upon admission were significantly higher in the mortality group [*p* ≤ 0.001, *p* = 0.008, *p* = 0.001, *p* = 0.004, and *p* = 0.036, respectively].

A higher NLR was significantly associated with fulminant sepsis and major arterial embolization [*p* = 0.001 and *p* = 0.028, respectively].

The receiver operating characteristic (ROC) curve of the NLR for predicting in-hospital mortality showed that an NLR > 8.085 had a 60% sensitivity and an 84.8% specificity for an association with in-hospital mortality [area under the curve = 0.729, 95% confidence interval (CI) 0.616–0.841; *p* = 0.001]. The ROC curve of the NLR for predicting severe sepsis showed that an NLR > 5.035 had a 71.8% sensitivity and a 68.5% specificity for predicting severe sepsis [area under the curve 0.685, 95% CI 0.582–0.733; *p* = 0.001].

The PLR showed no significant association with in-hospital mortality or in-hospital complications.

**Conclusion:**

A higher NLR, TLC, neutrophil percentage, creatinine level, and CRP level upon admission were associated with increased in-hospital mortality and morbidity in IE patients. Furthermore, a lower lymphocyte count/percentage and platelet count were strong indicators of in-hospital mortality among IE patients.

Calculation of the NLR directly from a CBC upon admission may assist in early risk stratification of patients with IE.

## Background

Infective endocarditis (IE) is an infection of the heart valves (native or prosthetic); large intrathoracic vessels; intracardiac structures, such as the interventricular septum, chordae tendineae, or mural endocardium; and intracardiac foreign bodies. Despite improvements in the diagnosis and treatment of IE, the in-hospital mortality and morbidity rates of this infection remain high [1–4].

Recently, with the growing understanding of the roles of various inflammatory markers in IE, studies have focused on new, widely available, and inexpensive inflammatory markers. Recent studies have revealed that various markers, including the WBC count, neutrophil-to-lymphocyte ratio (NLR), and mean platelet volume, are associated with IE and its prognosis [4–7].

The NLR, a novel marker, has been studied in various immunologic and infectious diseases as well as cardiologic disorders. The NLR is simply acquired using a complete blood count (CBC), which is the most commonly performed test in hospitals.

The NLR has been evaluated in various cardiac disorders, including atherosclerotic heart disease. Studies have revealed considerable prognostic value in addition to significant positive correlations. The predictive value of the NLR for peripheral arterial disease, calcific aortic stenosis, the presence, severity and extent of coronary artery disease, and many other cardiovascular disorders has been shown [8–15].

Platelets are well-established components of the hemostatic system. Recently, several additional functions of platelets have been demonstrated in the pathogenesis of various inflammatory diseases. The lymphocyte count is inversely correlated with inflammation. A lower lymphocyte count indicates an increased risk of inflammation, which correlates with cardiovascular disease burden and mortality [15, 16]. Therefore, the platelet-to-lymphocyte ratio (PLR) is positively correlated with inflammation and can therefore be a useful biomarker to predict the severity of inflammation.

Recently, the PLR was used as a worse prognostic marker for various cardiovascular conditions [17, 18]. However, very limited data are available regarding its predictive role in patients with IE [19]. In our study, we aimed to assess the relationship of the PLR and NLR that are obtained on admission with in-hospital morbidity and mortality in patients with IE, and we compared these two ratios.

## Methods

### Study design

This is a retrospective analytic study. Patients with definitive/possible IE according to the modified Duke criteria, as diagnosed by the Kasr Al Ainy IE Working Group from January 2011 through July 2016, were reviewed in this study. Only patients for whom full laboratory data were available were enrolled. The IE Working Group used the AHA/ACC guidelines and the ESC guidelines for the diagnosis and management of IE patients [1, 2].

At least three sets of blood cultures were collected for each patient prior to initiating antibiotics. Cultures were collected from different venepunctures, with the first and last samples drawn at least 1 h apart. Each blood culture set consisted of one BACTEC Plus Aerobic/F and one BACTEC Plus anaerobic/F culture vial (Becton Dickinson, Sparks, MD, UAE).

Surgically excised materials, including excised valves, vegetations, infected prostheses, aortic abscesses, and emboli, were submitted for Gram staining, potassium hydroxide preparation (KOH), histopathological examination, and microbial culture.

Serodiagnosis for the detection of antibodies specific to Brucella, Bartonella, and Coxiella and Aspergillus Galactomannan Antigen was performed routinely for all patients.

Trans-thoracic echocardiography (TTE) was performed within 24 h of admission. Transesophageal echocardiography (TEE) was performed, as indicated, within 48–72 h. All images were standardized according to the guidelines of the American Society of Echocardiography [20, 21].

All hematologic and biochemical data were obtained from venous blood samples drawn on the first day when patients were admitted to our hospital.

CBC hemoglobin, total leucocyte count (TLC), neutrophil, lymphocyte, and platelet counts were calculated using a Cell-Dyn 3700 automated cell counter. The PLR and NLR were directly obtained from the CBC on admission [22–24].

- Significant anemia was defined as a blood hemoglobin level < 10 mg/dl to avoid mild anemia that could be attributed to nutritional factors.
- Normal values of the TLC were defined as ranges from 4000 to 11000/c mm.
- Normal neutrophil percentage ranges were considered to be from 40 to 75%.
- Normal lymphocyte percentage ranges were considered to be from 20 to 45%.
- Normal platelet count ranges were considered to be from 150–400 × 10^3^/L.

C-reactive protein (CRP), serum creatinine, and rheumatoid factor levels were all assessed on admission and periodically across each patient’s in-hospital course.

Severe (fulminant) sepsis was defined as sepsis necessitating ventilation or vasopressor support.

### Statistical analysis

Data were coded and entered using the statistical package SPSS (Statistical Package for the Social Sciences) version 23. Data were summarized using the mean, standard deviation, median, minimum, and maximum for quantitative data and using frequencies (counts) and relative frequencies (percentages) for categorical data. Comparisons between quantitative variables were performed using the non-parametric Mann-Whitney test (Chan, 2003a) [25]. For comparing categorical data, the chi-square (χ²) test was performed. An exact test was used instead when the expected frequency was less than 5 (Chan, 2003b) [26]. A receiver operating characteristic (ROC) curve was constructed, and an area under the curve analysis was performed to detect the best cutoff values of the PLR and NLR for detecting mortality and complications. Multivariate logistic regression was performed to identify independent predictors of mortality and complications. *p* values less than 0.05 were considered as statistically significant [26].

### Approval

The study protocol was approved by the Kasr Al Ainy Ethical Committee.

## Results

We reviewed 217 patients who were admitted with a diagnosis of definitive/possible IE by the IE working group at Kasr Al Ainy Teaching Hospitals between January 2011 and July 2016. Complete hematological data were available for only 142 patients who were enrolled in the study.

Patients were young; the mean age was 30.95 ± 11.03 years (ranges 12–71). Males were more commonly affected than females (87 [61.3%] versus 55 [38.7%]). The median duration of hospitalization was 40 days (ranges 1–112 days).

### Predisposing risk factors

Predisposing factors for IE were rheumatic heart disease (RHD) in 74 patients (52.1%), prosthetic valve and intracardiac devices in 41 patients (28.9%), intravenous (IV) drug abuse in 27 patients (19.1%), congenital heart disease (CHD) in 12 patients (8.5%), and degenerative heart disease in 3 patients (2.1%). A history of previous IE was found in 8 patients (5.6%).

### Causative organisms

Causative organisms were identified by blood/tissue culture or serology in 85 patients (59.9%). The most common organisms were Staphylococci [40 patients (47.1%), 40% of them had methicillin-resistant *S. aureus* (MRSA)], followed by zoonotic organisms as a cause of IE in 14 patients (16.5%) [*Brucella* spp. in 8 patients, *Bartonella henselae* in 5 patients and *Coxiella burnetii* in one patient], and Streptococci (12 patients, 14.1%). Fungi were identified in 11 patients (12.9%).

### Laboratory parameters

CBCs were obtained on admission, and CBC parameters were analyzed. Serum creatinine, CRP, and rheumatoid factor levels were also analyzed. Patients were generally anemic with a mean hemoglobin level of 9.89 ± 1.82 gm/dL and a median of 9.75 gm/dL (range, 5.30–14.30 gm/dL). CRP levels were notably high, with a mean of 94.49 ± 74.62 mg/L. Serum creatinine levels were also elevated (mean, 2.19 ± 6.56 mg/dL).

Hematological and other laboratory characteristics on admission are shown in Table 1.

**Table 1:** Hematological and other laboratory characteristics of the patients

Mean	Standard deviation	Median		Minimum	Maximum
ESR	90.82	36.23	99.00	5.00	176.00
C-reactive protein (mg/L)	94.49	74.62	84.00	9.00	597.00
Serum creatinine (mg/dL)	2.19	6.56	1.00	0.39	12.00
Hemoglobin g/dL	9.89	1.82	9.75	5.30	14.30
TLC	11.63	5.55	10.70	3.70	35.60
Neutrophil percentage	72.17	14.58	75.50	26.00	97.00
Lymphocyte percentage	19.84	11.88	18.50	1.00	68.00
Lymphocyte count	1951.83	974.27	1801.00	177.00	6300.00
Platelet count	261.89	125.29	250.00	10.00	608.00
PLR	159.22	108.82	128.01	9.34	902.91
NLR	6.74	9.78	4.05	.40	97.00

*ESR* erythrocyte sedimentation rate, *CHF* congestive heart failure, *NYHA* New York Heart Association, *ICH* intracranial hemorrhage, *SAH* subarachnoid hemorrhage, *ARF* acute renal failure

### Clinical course and in-hospital outcome

Fifty-seven patients showed a good response to medical treatment, defined as improvement in the general condition of the patient, declining levels of inflammatory markers, and disappearance of fever in response to antimicrobial therapy without surgical intervention.

Cardiac surgery was indicated in 100 patients (70.4%); however, it was only performed in 74 patients; some of the patients died short out of having surgery due to severe morbid condition while some others were scheduled for elective surgery after remission of acute IE episode. The most common indications for surgery were congestive heart failure (CHF) in 47 patients (47% of the total patients with indications for surgery) followed by severe uncontrolled infection in 33 patients (33%).

Major complications, including CHF (NYHA class III–IV), cerebrovascular stroke (CVS), intracranial hemorrhage (ICH), acute renal failure (ARF) requiring dialysis, fulminant sepsis, and major arterial embolization, occurred in 107 patients of the whole group whether indicated for surgery or not. Table 2 shows the details of the complication incidences.

**Table 2:** Complication frequency in IE patients on admission and during hospitalization

	Number of patients (%) (total *N* = 142)*
CHF class NYHA III–IV	52 (36.6%)
Systemic and pulmonary embolization	90 (63.4%)
Fulminant sepsis	34 (23.9%)
Stroke	31 (21.8%)
ICH/SAH	11 (7.7%)
Cerebral mycotic aneurysm	9 (6.3%)
ARF/renal insufficiency**	40 (28.2%)
Splenic abscess/infarction	29 (20.4%)
Acute limb ischemia	4 (2.8%)
Other complications	3 (2.1%)

*CHF* congestive heart failure, *NYHA* New York Heart Association, *ICH* intracranial hemorrhage, *SAH* subarachnoid hemorrhage, *ARF* acute renal failure

*Most patients had more than one complication

**Renal insufficiency was defined as a serum creatinine level > 2 mg/dl

The overall in-hospital mortality was 21.1%. The main causes of in-hospital death were fulminant sepsis, severe heart failure, and surgery-related mortality (40%, 20%, and 16.7% of overall mortality, respectively).

### Predictors of in-hospital mortality

Fulminant sepsis, renal insufficiency (creatinine > 2 mg/dl), end-stage renal disease, splenic abscess/infarction, failure to respond to medical therapy alone, and major artery embolization were closely linked to mortality (*p* < 0.001, *p* < 0.001, *p* = 0.020, *p* = 0.054, *p* < 0.001, and *p* = 0.065, respectively). Clinical characteristics associated with increased in-hospital mortality are shown in Table 3.

**Table 3:** Clinical and hematological predictors of in-hospital mortality in IE patients

	Mortality (*N* = 30)	Survival (*N* = 112)	*p* value
Heart failure on admission	6 (20%)	10 (8.9%)	0.106
Fulminant sepsis	19 (63.3)	15 (13.4%)	< 0.001
Renal insufficiency creatinine > 2 gm/dl	17 (56.7%)	26 (23.2%)	< 0.001
End stage renal disease	12 (40%)	22 (19.6)	0.020
Splenic abscess/infarction	9 (30%)	20 (17.9)	0.054
Lung abscess	9 (30)	16 (14.3%)	0.103
Cerebral hemorrhagic infarction	4 (13.3%)	3 (2.7%)	0.036
Intracranial/subarachnoid hemorrhage	7 (23.3%)	4 (3.6%)	0.002
Embolization to different organs	23 (76.6%)	67 (59.8%)	0.065
Responded to medical treatment	3 (10%)	54 (48.2%)	< 0.001

Hemoglobin level was lower in the mortality group than in the non-mortality group (mean 9.31 ± 1.87 g/dL and 10.04 ± 1.79 g/dL, *p* = 0.076). A higher mean TLC on admission was noticed among the mortality group (14.6 ± 7.42 × 103/ml and 10.84 ± 4.66 × 103/ml, *p* = 0.008). The mean neutrophil percentage was significantly higher in the mortality group of patients (78.87% ± 15.11 to 70.37% ± 13.97, respectively, *p* = 0.001). A lower lymphocyte count/percentage and platelet count were strong indicators of the incidence of in-hospital mortality (*p* = 0.015, *p* < 0.001, and *p* = 0.001, respectively). CRP levels on admission were significantly higher (as a continuous variable) in the mortality group (the mean CRP level was 110 mg/L in the mortality group vs. 91 mg/L in the survival group, *p* = 0.036), and higher serum creatinine levels on admission and during hospitalization were significantly associated with an increased incidence of in-hospital mortality (*p* = 0.004 and *p*< 0.001, respectively).

**Fig. 1:**
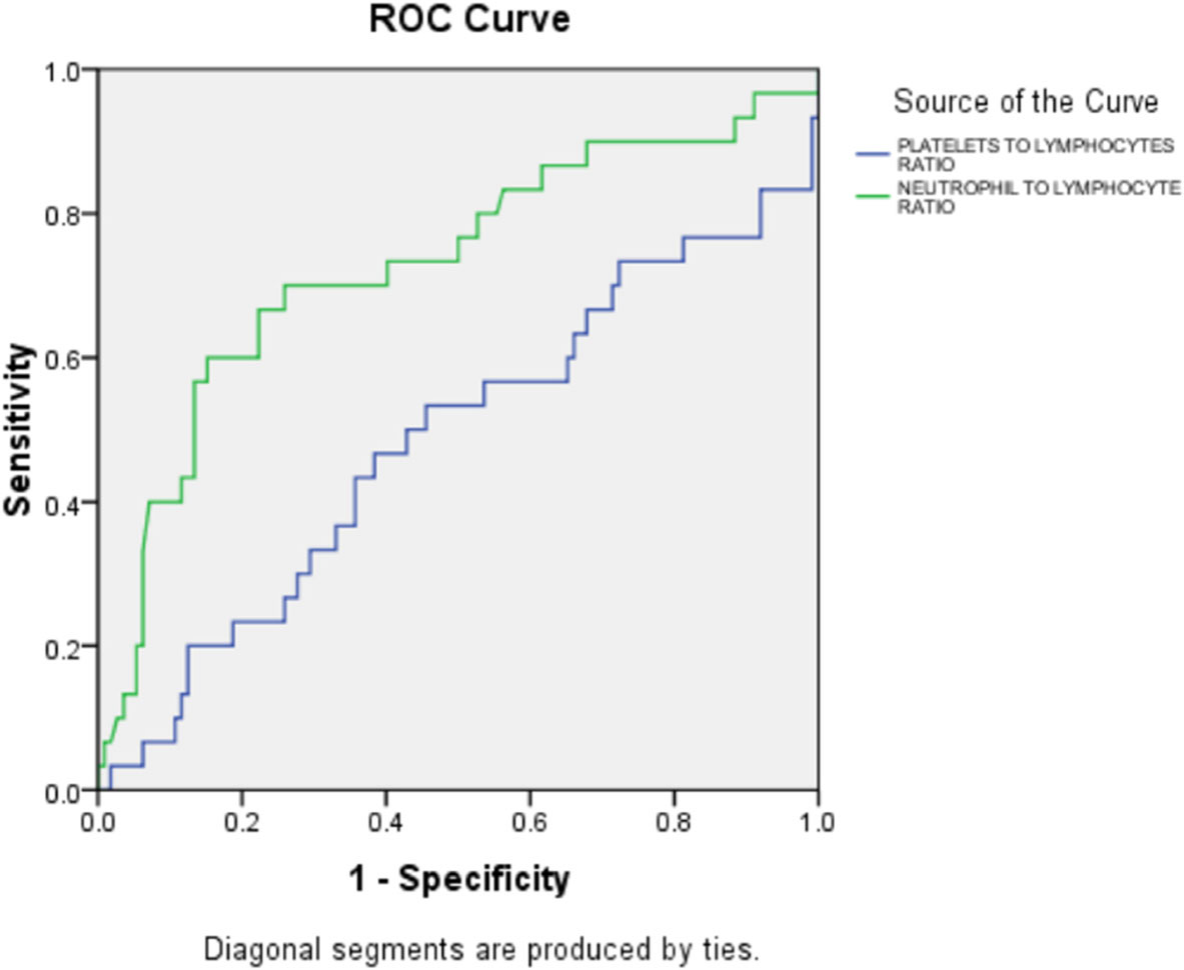
ROC curve for predicting mortality using the platelet-to-lymphocyte ratio and neutrophil-to-lymphocyte ratio

A higher NLR was significantly associated with an increased incidence of in-hospital mortality (*p* ≤ 0.001), and the ROC curve of the NLR for predicting in-hospital mortality is shown in Fig. 1. An NLR over 8.085 measured upon admission had a 60% sensitivity and an 84.8% specificity for predicting in-hospital mortality [area under the curve = 0.729, 95% confidence interval (CI) 0.616–0.841; *p* ≤ 0.001].

Laboratory characteristics associated with increased in-hospital mortality are shown in Table 4.

**Table 4:** Laboratory predictors of in-hospital mortality

	Mortality (*N* = 30)	Survival (*N* = 112)	*p* value
CRP on admission	110 (30–211)	91 (9–597)	0.036
Creatinine on admission	4.78 (0.39–12)	1.49 (0.4–15)	0.004
Highest creatinine level	3.99 (0.7–12)	2 (0.2–15)	< 0.001
Hemoglobin on admission	9.13 (5.3–12.7)	10 (5.9–14.3)	0.076
TLC on admission	14.60 (4–35.6)	10.84 (4.10–29)	0.008
Neutrophil percentage on admission	78.87% (27–97%)	70.37% (26–95%)	0.001
Lymphocyte percentage	14.2% (1–68%)	21.35% (2–52%)	< 0.001
Lymphocyte count	1569.53 (177–3672)	2054.2 (270–6300)	0.015
Platelet count	202 (10–587)	277.9 (56-608)	0.001
PLR	150 (9.24–395.5)	161.7 (26.9–9.2.9)	0.791
NLR	12.12 (0.4–97)	5.3 (0.52–47.5)	< 0.001

The number of vegetations was significantly associated with mortality; the higher the number of vegetations was, the higher the risk of the in-hospital mortality [*p* = 0.002]. Severe acute aortic regurgitation and prosthetic valve complications and MRSA or Candida infections were also associated with a high incidence of in-hospital mortality [*p* = 0.007, *p* = 0.030, and *p* = 0.030 or *p* = 0.028, respectively].

Through multivariate analysis, predictors of in-hospital mortality were a higher TLC on admission [odds ratio (OR) = 1.487; 95% CI, 1.173 to 1.863; *p* = 0.001], a low lymphocyte percentage on admission [OR = 1.207; 95% CI, 1.015 to 1.435; *p* = 0.033], and a low lymphocyte count on admission [OR = 0.998; 95% CI, 0.996 to 0.999; *p* = 0.008].

### Predictors of in-hospital morbidities

A higher NLR was significantly associated with an increased incidence of fulminant sepsis and major artery embolization (*p* = 0.001 and *p* = 0.028, respectively).

**Fig. 2:**
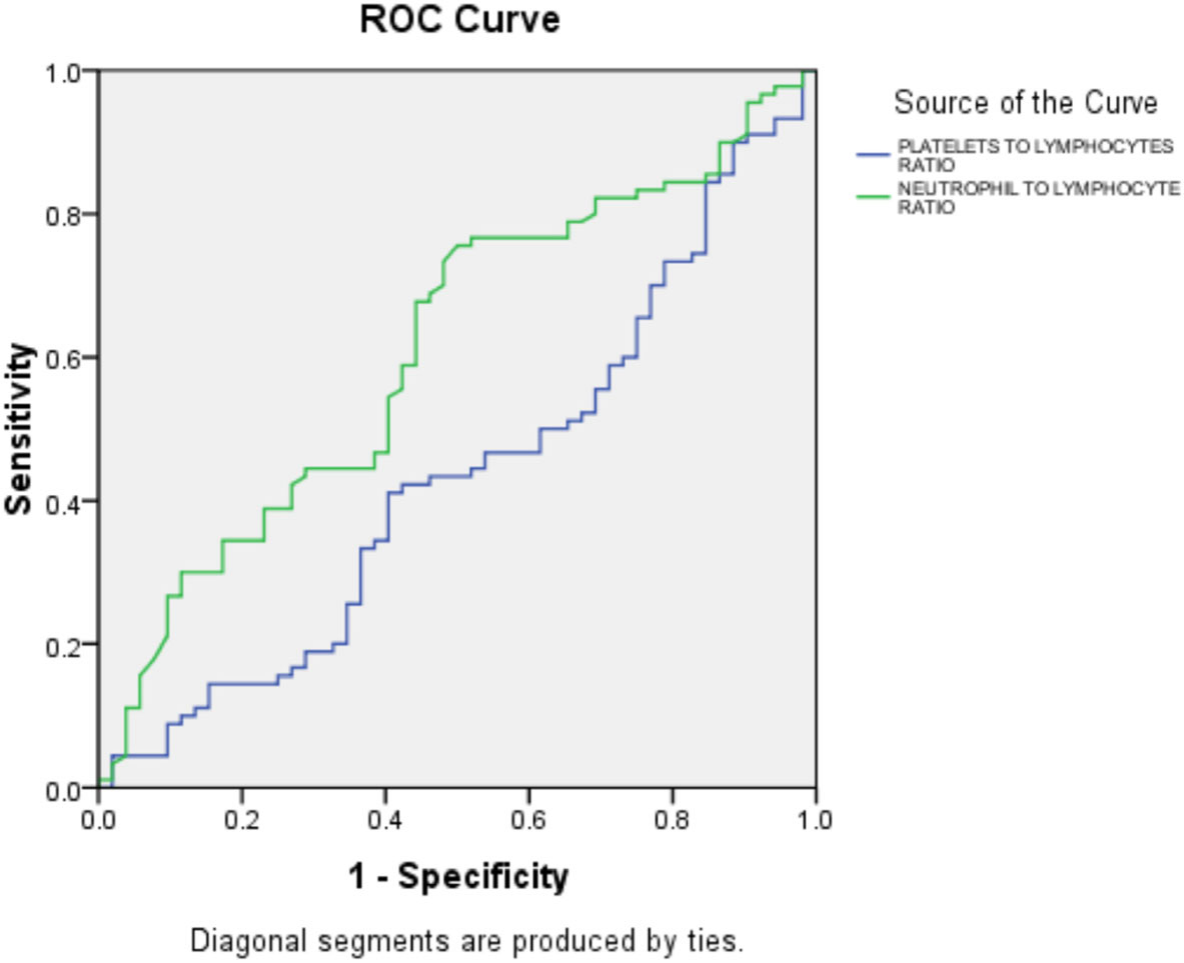
ROC curve to detect embolization using the platelet-to-lymphocyte ratio and neutrophil-to-lymphocyte ratio

The ROC curve of the NLR for predicting embolization to different organs is shown in Fig. 2. An NLR over 3.045 measured upon admission had a 73.3% sensitivity and a 51.9% specificity for predicting embolization [area under the curve 0.611, 95% CI 0.516–0.707; *p* = 0.028].

**Fig. 3:**
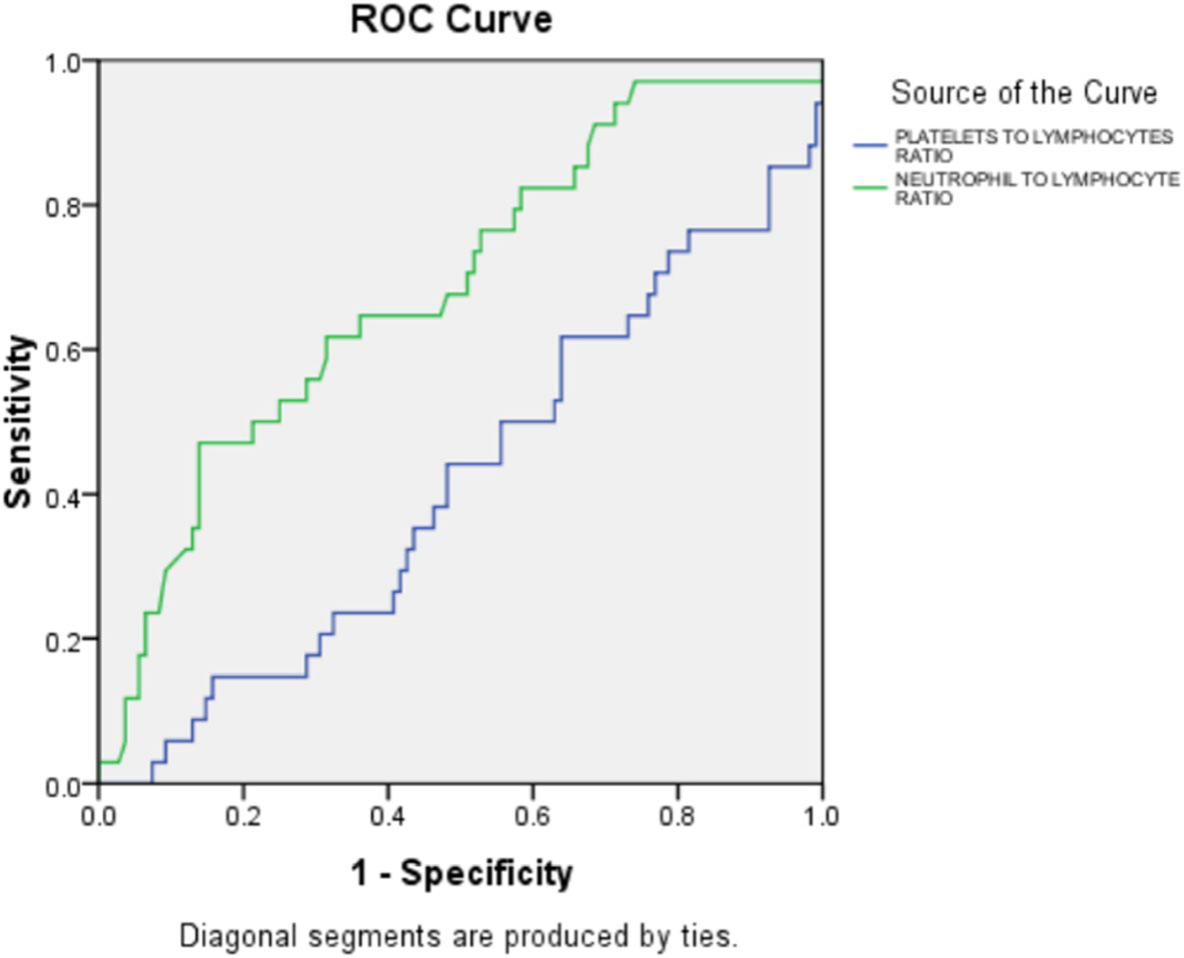
ROC curve to detect severe sepsis using the platelet-to-lymphocyte ratio and neutrophil-to-lymphocyte ratio

An NLR over 5.035 measured upon admission had a 71.8% sensitivity and a 68.5% specificity for predicting severe sepsis [area under the curve 0.685, 95% CI 0.582–0.733; *p* = 0.001]. The ROC curve of the NLR for predicting severe sepsis is shown in Fig. 3.

A higher CRP level on admission was also significantly associated with an increased incidence of CNS complications (*p* = 0.055).

While the PLR showed trends towards an increased incidence of in-hospital sepsis and major artery embolization (*p* = 0.136 and *p* = 0.186, respectively), there was no association between a higher PLR and in-hospital mortality in our patients.

## Discussion

IE is an infrequent yet life-threatening disease with considerable morbidity and mortality rates that have been unchanged for more than 3 decades [1, 2, 6, 27]. The in-hospital mortality rate of patients with IE varies from 15 to 30% [28–36].

Rapid identification of patients at highest risk of death may offer the opportunity to change the course of the disease (i.e., emergency or urgent surgery) and improve prognosis [37]. Current guidelines recommend additional protective measures in high-risk patients [1, 2]; however, identifying patients at greater risk in the early phases of disease is not easy because of the non-uniformity and complexity of IE.

The PLR and NLR are independent predictors of worse prognosis in many infectious and cardiovascular diseases [8–15, 17, 18]; however, a very limited number of studies have been conducted to evaluate the prognostic role of these markers in IE [5–7, 19].

The aim of this study was to determine the value of the PLR and NLR as predictors of in-hospital morbidity and mortality among IE patients.

We analyzed clinical, laboratory, and echocardiographic data and the clinical course throughout the entire period of hospitalization in a total of 142 consecutive patients with definitive/possible IE diagnoses. The PLR and NLR were obtained directly from a CBC obtained upon admission.

In our study, the mortality rate was 21% (30 patients), while complications of CVS, fulminant sepsis, acute heart failure, ARF, and embolization to different vital organs occurred in 38 (27%), 34 (24%), 32 (22.5%), 40 (28%), and 90 (63.4%) patients, respectively.

We found that an NLR over 8.085 measured upon admission had a 60% sensitivity and an 84.8% specificity for predicting in-hospital mortality among IE patients [area under the curve = 0.729, 95% CI 0.616–0.841; *p* ≤ 0.001]; the high cutoff value in our study compared to the cutoff value of 7 from the study by Turak et al. [6] may be due to delayed presentation from the onset of disease symptoms (the mean time to referral in our study was 43 days) or introduction of antibiotics before referral, which alters the TLC and the NLR.

Additionally, we found that a high NLR on admission was significantly associated with an increased in-hospital incidence of severe sepsis. The ROC curve of the NLR for predicting severe sepsis shows that an NLR over 6.19 measured upon admission had a 71.8% sensitivity and a 68.5% specificity for predicting severe sepsis [area under the curve = 0.685, 95% CI; 0.582–0.733, *p* = 0.001]. This is also a higher cutoff value than that in the report by Turak et al. [6].

A higher NLR measured upon admission was also associated with an increased incidence of embolization to different organs [*p* = 0.028].

In our study, the PLR showed no significant association with in-hospital mortality or the occurrence of in-hospital complications in IE patients. To the best of our knowledge, only one study has been conducted to evaluate the relationship between the PLR and in-hospital mortality in IE patients. Zencir et al. retrospectively analyzed the data from 59 patients diagnosed with definitive IE and found that a higher PLR on admission was associated with increased in-hospital mortality in patients with IE (*p* = 0.008) [19].

To date, very few reports have referred to a possible prognostic role of CBC parameters in identifying high-risk IE patients. Further studies might be needed to evaluate the prognostic significance of such parameters.

## Limitation of the study

The study was limited by being a retrospective study and by being carried out in a tertiary referral center where patients were referred late, which might have increased the incidence of morbidities and mortality.

## Conclusion

A CBC is an easy non-invasive test that is routinely performed on IE patients on admission and during follow-up. In our study, a higher NLR, TLC, and CRP and creatinine levels (> 1.99 mg/dL) obtained upon admission were associated with increased in-hospital mortality and morbidity in IE patients. Additionally, a lower lymphocyte count/percentage and platelet count were strong indicators of in-hospital mortality among IE patients.

Calculation of the NLR directly from a CBC obtained upon admission may assist in early risk stratification of patients with IE.

## Data Availability

All data generated or analyzed during this study are included in this published article.

## Abbreviations

ARF: Acute renal failure
CBC: Complete blood count
CHD: Congenital heart disease
CHF: Congestive heart failure
CI: Confidence interval
CRP: C-reactive protein
CVS: Cerebrovascular stroke
ICH: Intracranial hemorrhage
IE: Infective endocarditis
KOH: Potassium hydroxide preparation
MRSA: Methicillin-resistant *S. aureus*
NLR: Neutrophil-to-lymphocyte ratio
PLR: Platelet-to-lymphocyte ratio
RHD: Rheumatic heart disease
ROC curve: Receiver operating characteristic curve
TEE: Transesophageal echocardiography
TLC: Total leucocyte count
TTE: Trans-thoracic echocardiography
